# Olfactory Dysfunction in Patients With Coronavirus Disease 2019: A Review

**DOI:** 10.3389/fneur.2021.783249

**Published:** 2022-01-18

**Authors:** Guoli Wei, Jialin Gu, Zhancheng Gu, Cheng Du, Xiaofei Huang, Haiyan Xing, Lingchang Li, Aiping Zhang, Xingxing Hu, Jiege Huo

**Affiliations:** ^1^Affiliated Hospital of Integrated Traditional Chinese and Western Medicine, Nanjing University of Chinese Medicine, Nanjing, China; ^2^Jiangsu Province Academy of Traditional Chinese Medicine, Nanjing, China; ^3^Nanjing Lishui District Hospital of Traditional Chinese Medicine, Nanjing, China; ^4^Department of Oncology, Yangzhou University Medical College, Yangzhou, China; ^5^The Third Medical College, Nanjing University of Chinese Medicine, Nanjing, China; ^6^Affiliated Brain Hospital of Nanjing Medical University, Nanjing, China

**Keywords:** COVID-19, olfactory dysfunction, clinical characteristics, mechanism, treatment

## Abstract

The coronavirus disease 2019 (COVID-19) pandemic is wreaking havoc on public-health and economic systems worldwide. Among the several neurological symptoms of patients with COVID-19 reported in clinical practice, olfactory dysfunction (OD) is the most common. OD occurs as the earliest or the only clinical manifestation in some patients. Increasing research attention has focused on OD, which is listed as one of the main diagnostic symptoms of severe acute respiratory syndrome-coronavirus-2 infection. Multiple clinical and basic-science studies on COVID-19-induced OD are underway to clarify the underlying mechanism of action. In this review, we summarize the clinical characteristics, mechanisms, evaluation methods, prognosis, and treatment options of COVID-19-induced OD. In this way, we hope to improve the understanding of COVID-19-induced OD to aid early identification and precise intervention.

## Introduction

Coronavirus disease 2019 (COVID-19) is caused by severe acute respiratory syndrome-coronavirus-2 (SARS-CoV-2) infection. COVID-19 has wrought havoc on public-health and economic systems worldwide since January 2020. As of September 2021, more than 220 million people have been infected and 4.6 million people in 221 countries have died.

The typical clinical symptoms of COVID-19 are fever, cough, fatigue, headache, myalgia or joint pain, sore throat, olfactory dysfunction (OD), gustatory dysfunction (GD), and diarrhea (1–3). As one of the early and common symptoms of COVID-19, OD (classified as anosmia, hyposmia, or dysosmia) has been a focus of increasing research interest (4–6). Approximately 40–50% of patients report OD as the first or only symptom of COVID-19, which has led to the proposal that OD should be given high priority as a prodromal symptom of COVID-19 (7–12). In contrast to reports on infection by other viruses, OD is associated with a relatively good prognosis in COVID-19, which suggests distinct pathological mechanisms that require further clarification (13, 14).

In this study, we review the clinical characteristics, mechanisms, evaluation methods, prognosis, and treatment options of COVID-19-induced OD.

## Clinical Characteristics

### Epidemiology

A recent meta-analysis based on 83 studies provided high-quality evidence of a combined prevalence of OD in 47.9% of COVID-19 patients. Also, the prevalence of anosmia, hyposmia, and dysosmia was documented in 35.39, 36.15, and 2.53% of cases, respectively (15). OD is diagnosed more commonly among female patients and outpatients (16–18).

Following the first report of OD in 13.8% of patients with COVID-19 in China by Mao et al. (19), OD was identified as a widespread symptom in up to 60–90% of patients (20, 21). Following large-scale enrollment of participants, the reported prevalence of OD in patients decreased to 40–50% (22, 23). Due to the global nature of the pandemic, OD prevalence across countries is variable. For instance, studies have reported a prevalence of 13.8–67.2% in Asia (19, 24), 19–68% in North America (21, 25), 19.4–85.6% in Europe (20, 26), and 82.4% in Brazil (18). Interestingly, OD appears to be more prevalent in patients with mild-to-moderate COVID-19 than in those with severe disease (11, 27–30).

Notably, the variable geographic distribution of OD may be due to local differences in the emphasis placed on OD, the study cohort, assessment methods, and study design (9, 31). Moreover, self-reported tests may lead to underestimation of OD prevalence (28, 32–35). The number of patients with OD diagnosed using objective olfactory evaluations has been reported to be 2–3-times higher relative to that using self-reported (survey/questionnaire-reported) tests (23, 36, 37).

Different strains of SARS-CoV-2 can also cause different severities of OD. A systematic review incorporating scientific articles specific to the investigation of post-viral OD found that viral effects on the olfactory system differed according to the viral strain, along with alterations in or damage to components of the olfactory epithelium (OE) and/or olfactory bulb (38). Another systematic evaluation reported that the D614G virus mutation (compared with the D614 strain) increased OD prevalence in COVID-19 (39). In addition, the time of testing, ethnic/racial differences, age or sex, population density, and disease severity may be important contributory factors in prevalence variations among studies (17, 40–42).

### Clinical Presentation

Patients with COVID-19 may develop OD suddenly without other respiratory symptoms, such as nasal obstruction, rhinorrhea, and sore throat (1, 43, 44). Lechien et al. examined 1,363 ambulatory and hospitalized patients, 48.5–64.4% of whom presented with a sore throat, nasal obstruction, and rhinorrhea, which was lower than the reported prevalence of OD (81.6%) (27). Significant correlations were not observed between the severity of OD and other nasal symptoms (35).

As a common peripheral neuropathy of COVID-19, OD is closely associated with GD, with several reports of co-accompanying symptoms of OD and GD (45–47). Kaye et al. proposed that GD is a sequela of OD (48). In contrast, Singer-Cornelius et al. suggested, based on a lack of significant associations between the two conditions in objective tests, that GD and OD are two independent symptoms (37). A negative correlation has been demonstrated between OD and post-admission severity and mortality of COVID-19 in several studies (49, 50), in direct contrast to a report on clinical outcomes by Tabari et al. (51). Speth et al. showed that in addition to GD, OD severity was significantly associated with depression and anxiety (52). Interestingly, one study reported rare cases of “phantom OD” after a COVID-19 diagnosis, with a small proportion of cases being associated with objective hyposmia, whereas other patients were normosmic (53). An increasing number of studies have focused on the quantitative evaluation of olfactory function to identify the asymptomatic COVID-19 carriers (54, 55). A standardized quantitative test for an olfactory function could be a cost-effective and high-impact method for widespread screening and monitoring of COVID-19 (56).

### Risk Factors

#### Sex

Female patients infected with COVID-19 are more likely to develop OD. Jain et al. identified 42 women (49.6%) and 50 men (23.6%) with OD among 410 COVID-19 cases (14). Similar clinical outcomes have been reported in studies by Lechien et al. and Speth et al. (17, 57). In contrast, Meini et al. demonstrated that OD occurred less frequently, but lasted longer, in female patients (58). Interestingly, women were shown to be more susceptible to OD (in particular, hyposmia and anosmia) than men (20). This sex-based difference may be attributed to the different inflammatory processes between men and women.

#### Age

Olfactory dysfunction occurs frequently in patients with COVID-19, especially in the younger population. A high-quality meta-analysis showed a significant negative association between age and OD, either alone or combined with GD (28). In addition, increasing the mean age of patients with COVID-19 was found to be closely associated with a reduced prevalence of OD (23). However, in a 6-month follow-up study on 169 patients with mild-to-moderate COVID-19, Cristillo et al. showed that patients with long-term OD (identified *via* objective assessment) were older (68.2 years) compared with those with normal olfactory function (58.2 years) (59). They also found a 2-fold increase in OD risk in individuals aged >65 years and a 3-fold increase in those aged >75 years (59).

#### Ethnicity and Region

The prevalence of OD is higher in Caucasians, with reports of 3–6-times greater prevalence relative to that in Asians and African–Americans (28, 60, 61). In addition to racial differences, the prevalence of chemosensory dysfunction (CD) has been reported to be higher in cohorts from Europe, North America, and the Middle East (19–98%) compared with that in an Asian cohort (11–15%) (5). An international multicenter study reported OD prevalence among Chinese, German, and French patients to be 32, 69, and 49%, respectively (9). The susceptibility of individuals from distinct regions to SARS-CoV-2 infection may explain (at least in part) the racial differences in prevalence (62).

#### Other Potential Risk Factors

Large-scale clinical samples are required for the identification of the potential risk factors for OD. The most common comorbidities reported in patients with OD are obesity, hypertension, diabetes mellitus, and cardiovascular disease (63–65). Genetic pathways cannot be ignored in COVID-19 and its complications. A study on 3,261 Twins UK volunteers reported 19% heritability of OD (66). Tobacco smoking, asthma, allergic rhinitis, chronic obstructive pulmonary disease, muscle/joint pain, and hypothyroidism are also potential risk factors for OD (22, 67–69). However, large-scale clinical data are lacking, which hampers the drawing of conclusions from studies. Vaira et al. reported an increased likelihood of hyposmia with fever, chest pain, and sputum generation, but not anosmia or an olfactory disturbance (35). Interestingly, studies showed higher viral loads in CD patients with COVID-19 than those in non-CD patients (70), but individual viral loads were not associated with the prevalence, severity, or recovery from OD in subsequent investigations (71, 72). In addition, a study of OD outside of the COVID-19 pandemic found a low educational level, reduced self-perceived olfactory function, and exposure to toxic substances/irritants to be potential risk factors for OD as well, findings that could help to identify the new potential risk factors of OD in patients with COVID-19 (73).

## Mechanisms of Covid-19-Induced OD

**Figure 1:**
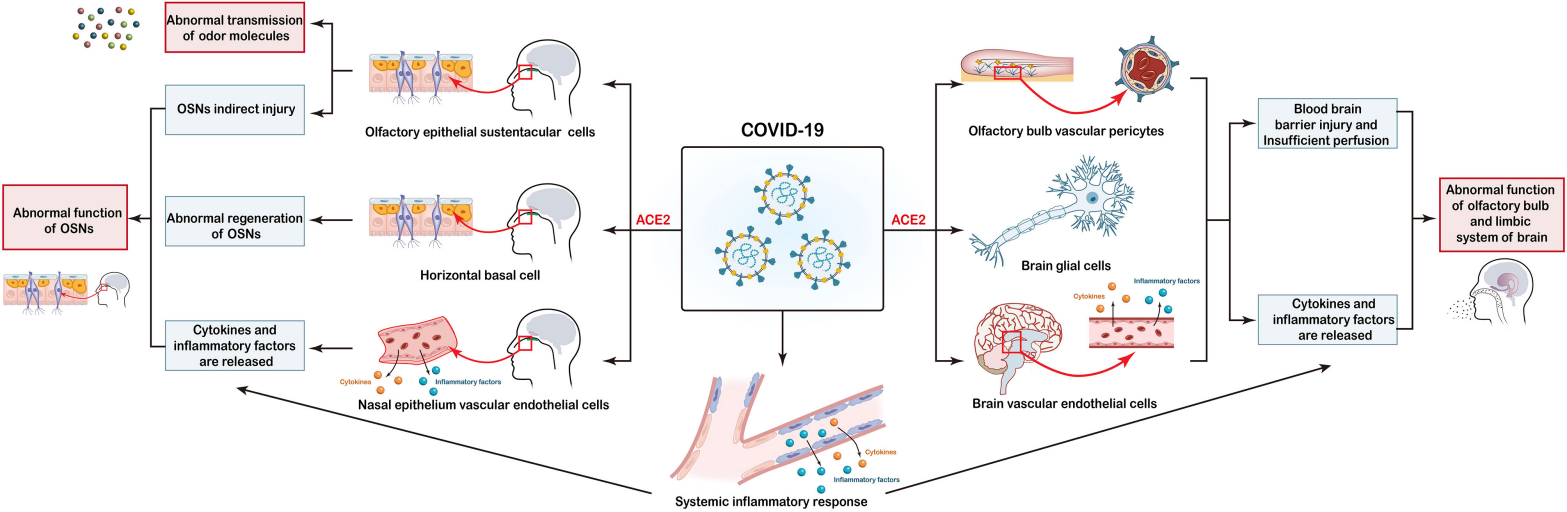
Potential mechanisms of coronavirus disease 2019 (COVID-19)-induced olfactory dysfunction. (i) COVID-19 directly affects sustentacular (SUS) cells through interactions with the ACE2 receptor, thereby leading to abnormal transmission of odor molecules. (ii) Injury to SUS cells, horizontal basal cells (HBCs) as well as the release of cytokines and proinflammatory factors in the systemic and local nasal epithelium lead to abnormal function of olfactory sensory neurons (OSNs). (iii) Olfactory-bulb vascular pericytes, glial-cell injury, and release of cytokines and proinflammatory factors into the systemic and local brain trigger abnormalities in functions of the olfactory bulb and limbic system of the brain.

Infections by several types of viruses cause OD, but the high prevalence and relatively short recovery time of OD induced by SARS-CoV-2 infection suggest a distinct mechanism (20, 74). Angiotensin-converting enzyme 2 (ACE2) is a functional receptor for SARS-CoV-2. It facilitates the entry of SARS-CoV-2 using the serine protease TMPRSS2 to “prime” the “spike” protein (75–77). ACE2 is associated significantly with OD under conditions of SARS-CoV-2 infection (77, 78). Although the underlying mechanisms are incompletely understood, data from several studies have provided novel insights. The current hypothesis is that SARS-CoV-2 causes OD through multiple pathways, as depicted in Figure 1.

### Central Nervous System Injury

The olfactory bulb, olfactory field, and limbic areas (e.g., olfactory media and hippocampus) are mainly involved in olfactory formation in the central nervous system (CNS). Lu et al. reported significantly higher bilateral gray-matter volumes in olfactory cortices, hippocampi, insulas, and left rolandic operculum, and the general decline of diffusivity in white matter, in patients with COVID-19 (70). Coronaviruses can invade the CNS *via* a transneuronal or hematogenous route. One *post mortem* study demonstrated the presence of SARS-CoV-2-specific antigens and RNA in the cerebrum of infected patients (79). Meinhardt et al. demonstrated the presence of SARS-CoV-2 RNA and protein in anatomically distinct regions of the nasopharynx and brain. They proposed that SARS-CoV-2 could enter the CNS through the neural–mucosal interface of the olfactory mucosa (80). Such new findings add to the understanding of the interactions between SARS-CoV-2 and the brain. However, whether the viral invasion of the CNS *via* the olfactory system is responsible for COVID-19-induced OD is not clear (81). It was initially hypothesized that SARS-CoV-2 might invade the olfactory neurons of the CNS directly, thereby leading to OD. However, subsequent studies showed that ACE2 was not expressed in olfactory neurons of the olfactory bulb (79, 82). Therefore, SARS-CoV-2 is believed to cause CNS injury through other mechanisms to lead to OD.

Central nervous system injury may be attributable to two main factors. First, ACE2 is expressed in many neurons (excitatory and inhibitory) and some non-neuron cells (mainly astrocytes, oligodendrocytes, endothelial cells, and olfactory-bulb vascular pericytes) in the human middle temporal gyrus and posterior cingulate cortex (78, 79, 82–85). After entry into the body, coronaviruses invade these cells directly and cause damage, produce proinflammatory mediators, and destroy the blood-brain barrier. Injury to blood vessels around the olfactory bulb leads to insufficient perfusion and damage to neurons which, ultimately, affects the function of the olfactory center and results in OD (84, 86–88). Second, SARS-CoV-2 infection triggers dysfunction of the immune system and overproduction of numerous cytokines, such as interleukin-1, interleukin-6, interleukin-12, interferon-γ, and tumor necrosis factor-α (89–91). Cytokine-driven injury and immune-mediated toxicity may destroy the integrity of the blood-brain barrier and cause secondary injury (92). Cytokines may also be directly neurotoxic, mediate, or even inhibit injury to cells of the CNS, either alone or in tandem with other factors (93).

### Peripheral Nervous System Injury

Peripheral tissues related to the olfactory system are distributed mainly in the nasal epithelium, which is classified into the respiratory epithelium (RE) and OE. The primary function of the RE is to humidify air as it enters the nose. The OE contains OSNs, SUS cells, and basal cells. The OE is located in the nasal cavity, where neurons are exposed to the external environment. The OE is responsible for the detection and transmission of odor signals to the brain.

In the early stages of SARS-CoV-2 infection, viral RNA can be detected readily in the upper respiratory tract, which suggests active infection and replication in this region (94). Single-cell RNA sequencing datasets from healthy individuals generated by the Human Cell Atlas Consortium revealed differential expression of ACE2 and TMPRSS2 protease in respiratory and intestinal epithelial cells, with the highest levels being observed in the nasal epithelium (95). Immunostaining of tissues from the human nasal epithelium showed significantly higher expression of ACE2 in the OE relative to that in the RE. However, ACE2 was not detected in olfactory neurons, a finding that was confirmed in a mouse model (82, 96, 97). Nevertheless, recently Cecchini et al., in addition to the clear high expression of ACE2 in the SUS cells of olfactory mucosa samples from humans, showed a sharp dot-like ACE2 expression in olfactory neurons, suggesting possible direct damage to neurons (98). In addition, a recent *post mortem* study clearly showed SARS-CoV-2 in human olfactory neurons (80).

Peripheral nervous system injury may be attributable to three main mechanisms. The first potential cause is the injury to olfactory epithelial SUS cells. In the physiological state, SUS cells have two main roles. First, SUS cells form the mucus layer of the OE to facilitate the molecular movement of odor molecules and make contact with the cilia of olfactory neurons. Second, SUS cells are close to olfactory neurons and provide structural support for sensory neurons. These cells have specific characteristics (e.g., phagocytosis of potentially harmful substances), a detoxification role, and maintain a local balance of salt and water (99, 100). SARS-CoV-2 affects SUS cells directly through interactions with the ACE2 receptor and TMPRSS2, resulting in impaired or temporary loss of SUS-cell function and, ultimately, abnormal transmission of odor molecules and direct/indirect damage to OSNs (82, 101).

Second, injury to HBCs may have an underlying role. Basal cells of the OE are subdivided into two types: globose basal cells (GBCs) and HBCs. GBCs are primarily responsible for regenerating OSNs during normal epithelial turnover. HBCs can aid the proliferation of epithelial cells and are activated as stem cells upon tissue injury, thereby supplementing GBC activity (102–104). In basal cells, expression of ACE2 and TMPRSS2 is detected only in HBCs, which suggests that SARS-CoV-2 can infect HBCs directly, resulting in abnormal regeneration of OSNs (82).

Third, SARS-CoV-2 infection can lead to vascular endothelial damage and abnormal production of cytokines and proinflammatory mediators. These actions affect CNS function but also cause secondary damage to nasal epithelial cells, thereby resulting in indirect effects upon olfactory function (13, 84, 87, 88, 105).

### Evaluation Methods

Two evaluation methods of COVID-19-associated OD are recommended: (i) questionnaire survey for subjective evaluation; (ii) olfactory sensitivity test for objective evaluation. Imaging and invasive endoscopy for OD alone are not advised (106).

### Subjective Assessment

The methods employed for subjective evaluation are visual analog scales and questionnaires. Visual analog scales are simple and effective tools to evaluate the presence and degree of olfactory function (9). The short version of the Questionnaire of Olfactory Disorders-Negative Statements (sQOD-NS) is a validated olfactory-specific quality of life (QoL) survey that quantifies patient perception of olfactory function. It is used widely in clinical studies on COVID-19 for people with olfactory dysfunction (10, 20, 107, 108). Despite the wide use of sQOD-NS to evaluate the QoL and mood impacts of OD from a subjective viewpoint, one main drawback is a lack of relevance to COVID-19 characteristics. To improve the specificity for COVID-19, the COVID-19 Task Force of Young Otolaryngologists of the International Federation of Oto-rhino-laryngological Societies (which includes otolaryngologists from North America, Europe, and Asia) has developed a questionnaire composed of the relevant epidemiological and clinical features. This version can be applied to determine the variations, timings, and associated symptoms of OD, thereby providing a reliable instrument for assessing COVID-19-induced OD (20).

Olfactory function is difficult to discriminate subjectively. For a quantitative assessment, also carrying out a validated test (i.e., assessment of hyposmia and anosmia) is beneficial. For qualitative evaluation, questionnaires assessing the frequency of occurrence, intensity, social impact, and visual scales are meaningful (i.e., assessment of phantosmia and parosmia) (109).

### Objective Assessment

The “Sniffin' Sticks” test is a nasal chemosensory assay that utilizes a pen-like odor-distribution device. This test has increased the prevalence of detection of OD in COVID-19 (110, 111). The Sniffin' Sticks test can reflect the level of olfactory function of individuals objectively. However, similar to sQOD-NS, owing to a lack of specificity for COVID-19, stage-specific analysis in conjunction with the disease stage and treatment plan is difficult (111).

Other olfactory sensitivity tests for OD in COVID-19 include the University of Pennsylvania Smell Identification Test, Toyota & Takagi Olfactometer, Cross-Cultural Smell Identification Test, Connecticut Chemosensory Clinical Research Center Test, and Brief Smell Identification Test (37, 112, 113). However, these assays must be developed further and optimized for more specific detection of COVID-19-induced OD.

### Prognosis

Coronavirus disease 2019-induced OD is reversible carries a good prognosis and, in general, is characterized by a high chance of recovery. Overall, 48.6–89% of COVID-19 patients with OD experience complete remission or improvement after 4 weeks of follow-up (63, 114, 115). In a recent meta-analysis, the mean recovery period of COVID-19-induced OD was 7.21 ± 12.93 days (22). Significant differences in the prevalence of OD recovery were not observed between the sexes. However, the recovery time for OD was longer in female and older patients (58, 116). Higher susceptibility in a European population was additionally associated with a longer recovery period. A European study involving 2,581 patients with COVID-19 reported a mean OD recovery period of 21.6 ± 17.9 days (27). However, several patients experienced slow recovery or persistent OD, which led to significant negative effects on QoL and morbidity in the form of nutritional disruption, social anxiety, or depression (117–119). Several factors influence rehabilitation from OD: region, ethnicity, sex, age, duration, and method of treatment. Further research is essential for identifying populations with a poor prognosis to facilitate efficacious interventions at early stages.

### Treatment Options

#### Olfactory Training

Olfactory training (OT) is an efficacious method to manage OD caused by various factors. Meta-analyses have shown that OT can significantly improve OD caused by viral infections (120–122). OT is also recommended for improving OD caused by COVID-19 (112, 113, 122). Clinical OT methods include classical olfactory training (COT) and modified olfactory training (MOT). COT involves exposure to four odors (phenyl ethyl alcohol, eucalyptol, citronellal, and eugenol) two times a day for 5 min each time for ≥12 weeks (123). MOT is based on the classic 12-week COT with added exposure to the second group of spices (menthol, thyme, tangerine, and jasmine) for 12 weeks subsequently and a third group of spices (green tea, bergamot, rosemary, and gardenia) for the final 12 weeks, thereby comprising a total treatment cycle of 36 weeks (124). The indications, treatment times, and specific effects of OT for patients with COVID-19-induced OD have yet to be established, and high-quality prospective studies are required to provide more concrete evidence.

#### Oral or Topical Corticosteroids

Oral or topical corticosteroids have been shown to improve olfactory function in patients. However, early studies included patients with localized nasal inflammation, such as rhinitis and sinusitis (125–128). Moreover, other studies suggested no significant effects of oral or topical corticosteroids on OD (129, 130). A recent systematic review revealed a lack of high-quality studies demonstrating the efficacy of oral or topical corticosteroids on OD unrelated to sinonasal disease (131). In contrast to other types of viral infections, OD in COVID-19 is not correlated significantly with nasal symptoms, such as nasal obstruction or rhinorrhea. Therefore, routine use of oral or topical corticosteroids for OD in COVID-19 is not recommended. For patients with inadequate OT or those participating in clinical studies, oral or topical corticosteroid therapy should be based on knowledge of the underlying disease, existing comorbidities, imaging findings, and adequate risk-benefit assessment, particularly the impact of short-term corticosteroids (112, 113).

#### Other Drugs

Numerous drugs exert potential therapeutic effects on OD: vitamin A, theophylline, intranasal sodium citrate, caroverine, alpha-lipoic acid, minocycline, zinc sulfate, and ginkgo biloba (112, 113, 132, 133). However, except for one case report on vitamin A, clinical studies on their efficacy in COVID-19-induced OD are lacking, so most of these drugs are not recommended for routine use (134).

## Conclusions

Olfactory dysfunction prevalence in COVID-19 is high, especially in young and middle-aged individuals, women, patients of European and American Caucasian descent, and those with mild disease. As one of the prodromal clinical symptoms of COVID-19, the inclusion of OD in the screening test is recommended. Current findings suggest that OD in COVID-19 is not caused by direct damage to OSNs but instead is a result of indirect damage to central and peripheral OSNs triggered by multiple pathways, even if the question is still open. Despite the high prevalence of COVID-19-induced OD, efficacious intervention strategies are lacking. OT is considered an efficacious and safe treatment option. Further studies are required to validate its efficacy and indications for oral or topical use of corticosteroids. Considering that COVID-19-induced OD carries a good prognosis and a short recovery period, the focus should be on identifying patients with a poor prognosis who may benefit from early intervention to avoid complications such as OD-induced depression and anxiety.

## Author Contributions

GW was involved in writing the original draft and methodology. JG and ZG were involved in writing the original draft. XHua performed conceptualization and writing the original draft. HX performed conceptualization. LL and CD were involved in writing the review and editing. JH and XHu supervised the manuscript. All authors contributed to the article and approved the submitted version.

## Funding

This study was supported by the National Natural Science Foundation of China (82004339), Project of National Clinical Research Base of Traditional Chinese Medicine in Jiangsu Province (JD2019SZXYB02), Jiangsu Province TCM Leading Talent Training project (SLJ0211), and Scientific Research Project of Jiangsu Provincial Health Commission (H2019095).

## Conflict of Interest

The authors declare that the research was conducted in the absence of any commercial or financial relationships that could be construed as a potential conflict of interest.

## Publisher's Note

All claims expressed in this article are solely those of the authors and do not necessarily represent those of their affiliated organizations, or those of the publisher, the editors and the reviewers. Any product that may be evaluated in this article, or claim that may be made by its manufacturer, is not guaranteed or endorsed by the publisher.

## Abbreviations

COVID-19: Coronavirus disease 2019
SARS-CoV-2: severe acute respiratory syndrome-coronavirus-2
OD: olfactory dysfunction
GD: gustatory dysfunction
ACE2: angiotensin-converting enzyme 2
SUS: sustentacular
CNS: central nervous system
PNS: peripheral nervous system
HBC: horizontal basal cell
OSN: olfactory sensory neuron
RE: respiratory epithelium
OE: olfactory epithelium
CD: chemosensory dysfunction
GBC: globose basal cell
QoL: quality of life
sQOD-NS: short version of the Questionnaire of Olfactory Disorders-Negative Statements
OT: olfactory training
COT: classical olfactory training
MOT: modified olfactory training.
